# Exercise and the Brain: Lessons From Invertebrate Studies

**DOI:** 10.3389/fnbeh.2022.928093

**Published:** 2022-06-28

**Authors:** Varvara Dyakonova, Maxim Mezheritskiy, Dmitri Boguslavsky, Taisia Dyakonova, Ilya Chistopolsky, Etsuro Ito, Igor Zakharov

**Affiliations:** ^1^Koltzov Institute of Developmental Biology of the Russian Academy of Sciences, Moscow, Russia; ^2^Department of Biology, Waseda University, Tokyo, Japan

**Keywords:** intense locomotion, motor performance, cognitive functions, desicion making, orientation, nerve regeneration, invertebrate model organisms, learning and memory

## Abstract

Benefits of physical exercise for brain functions are well documented in mammals, including humans. In this review, we will summarize recent research on the effects of species-specific intense locomotion on behavior and brain functions of different invertebrates. Special emphasis is made on understanding the biological significance of these effects as well as underlying cellular and molecular mechanisms. The results obtained in three distantly related clades of protostomes, Nematodes, Molluscs and Artropods, suggest that influence of intense locomotion on the brain could have deep roots in evolution and wide adaptive significance. In *C. elegans*, improved learning, nerve regeneration, resistance to neurodegenerative processes were detected after physical activity; in *L. stagnalis*—facilitation of decision making in the novel environment, in *Drosophila*—increased endurance, improved sleep and feeding behavior, in *G. bimaculatus*—improved orientation in conspecific phonotaxis, enhanced aggressiveness, higher mating success, resistance to some disturbing stimuli. Many of these effects have previously been described in mammals as beneficial results of running, suggesting certain similarity between distantly-related species. Our hypothesis posits that the above modulation of cognitive functions results from changes in the organism’s predictive model. Intense movement is interpreted by the organism as predictive of change, in anticipation of which adjustments need to be made. Identifying the physiological and molecular mechanisms behind these adjustments is easier in experiments in invertebrates and may lead to the discovery of novel neurobiological mechanisms for regulation and correction of cognitive and emotional status.

## Introduction

Physical exercise is one of the most powerful forms of behavioral modulation of cognitive functions in mammals: humans and rodents (Beckett et al., 2015; da Costa Daniele et al., 2020). It is known to activate neurogenesis, facilitate learning and decision-making, ameliorate the effects of stress, decrease anxiety and depression. Several brain neuromodulatory and neurotrophic systems were reported to participate in beneficial effects of exercise (Heijnen et al., 2016). Epigenetic changes in brain gene expression are repeatedly documented in rats and mice and extend to next generations of trained animals (Yang et al., 2021). The biological meaning and the evolutionary origin of these profound effects of intense locomotion on brain function, however, remains unclear today. Recently emerging data from research on distantly-related invertebrates suggest that these effects could have taken place already at the early stages of animal evolution and may have some universal adaptive significance. These discoveries also open new perspectives for studying the cellular and molecular mechanisms of the underlying influence of exercise on brain function, as many invertebrates are excellent experimental models.

In this review, we will summarize recent research on the effects of species-specific intense locomotion on behavior and brain functions of different invertebrates. Special emphasis will be made on possible biological significance of these effects as well as underlying cellular and molecular mechanisms.

## Nematodes

### Caenorhabditis elegans

Different regimes of swimming which has a greater energy cost than crawling (Laranjeiro et al., 2017), were used in *C. elegans* exercise protocols (Chuang et al., 2016; Chaudhari and Kipreos, 2017; Laranjeiro et al., 2017, 2019; Hartman et al., 2018; Hughes et al., 2019; Kumar et al., 2021). Already a single 90 min session induced antioxidant defense, reduced glucose metabolism and increased fat metabolism in *C. elegans* (Laranjeiro et al., 2017). Metabolic genes expression remained in this state for up to four hours after the exercise. At the same time, the pattern of genes expression was different from those produced by nonspecific stress (Laranjeiro et al., 2017). Regular swimming (in up to 6 days) changed muscle mitochondrial respiration and morphology, protected against mitotoxicity (Chaudhari and Kipreos, 2017; Hartman et al., 2018; Laranjeiro et al., 2019), improved locomotor performance (Laranjeiro et al., 2019) and decreased age-related (Chuang et al., 2016) or Duchenne muscular dystrophy (Hughes et al., 2019). The above exercise benefits may indirectly affect the nervous system by improving nutrient and energy supply and protecting from oxidative stress.

Direct evidence of swimming-induced changes in the nervous functions have also been obtained in *C. elegans*. First, better learning abilities (by an average 35%) in associative odor-food paradigm were detected after four days of swimming (Laranjeiro et al., 2019), however the short-term memory of animals undergoing the exercise did not differ significantly from the control. Second, swimming had a protective and long-lasting effect against a number of neurodegenerations, namely, tauopathy, Alzheimer’s disease, Huntington’s disease (Laranjeiro et al., 2019). For example, it caused an increase in the velocity of worms modified for aggregating Tau, and a decrease in the number of gaps in their GABAergic motor neurons compared to the control tau-aggregating animals. Third, a single swimming session following the axotomy of an identified neuron accelerated axon regeneration and behavioral recovery in larva, while in adult worms, several exercise sessions were needed to obtain the same effect (Kumar et al., 2021). The key molecule required for swimming-induced axon regeneration is identified to be, namely, the cellular energy sensor AAK-2/AMPK (Kumar et al., 2021). This is an interesting finding which may help understand molecular interplay in several interacting phenomena such as exercise, neuronal excitation and plasticity, insulin signaling and life span. AAR-2/AMPK is on the crossroad of these pathways. Its activation dependent on insulin signaling increases the lifespan in *C. elegans* (Park et al., 2020). Mid-life 15% better survival was observed in exercised worms as well (Laranjeiro et al., 2019), whether activation AAR-2/AMPK is responsible for this effect of exercise remains unknown.

## Molluscs

### Lymnaea stagnalis

Facilitation of decision-making by preceding intense locomotion has been reported in the mollusc *Lymnaea stagnalis* (Korshunova et al., 2016; Aonuma et al., 2020), one of the most popular organisms in cellular neuroscience due to its large and colored neurons (Rivi et al., 2019). The exercise included two hours of crawling in conditions of low water, which requires stronger muscular contractions than aquatic locomotion. Beneficial effect of exercise on decision-making was observed immediately after the exercise and 2 h later, after a period of rest in normal aquatic conditions. The behavioral paradigm used in these studies included orienting behavior in a novel completely dry arena with a gradient of light. Snails placed in the central part of the arena needed to make a decision which way to go to avoid the potentially dangerous situation of being out of water. They performed rotational moving at the beginning but then switched to a goal-oriented locomotion characterized by a higher speed and the lack of rotations. The “goal” was either the light side (ca 75% of snails) or the opposite, the dark wall (ca 25%; Korshunova et al., 2016; Vorontsov and Dyakonova, 2017). Previous exercise did not change the above proportion of choices, however, the trained animals started to act in the arena earlier, made fewer turns, had higher speed of locomotion and reached the border of the arena more quickly. To exclude the possible impact of stress, three different stressors were tested, namely, novelty, turbulent flow and occasional shell breaking (Korshunova et al., 2016; Aonuma et al., 2020). These stressors produced effects different from those of intense crawling.

Searching for cellular correlates of the above effects of terrestrial crawling resulted in several interesting new insights (Dyakonova et al., 2019). First, 2 h of crawling appear to change the biophysical properties of serotonergic neurons, isolated from the pedal cluster A (PeA; Dyakonova et al., 2019). The cluster controls locomotion by delivering serotonin directly to the sole. It is also known for prominent volume secretion of serotonin within the pedal ganglia (Chistopol’skii and Sakharov, 2003) and into the hemolymph (Dyakonova et al., 2015a, b). Second, 2 h of rest following exercise are still revealed in an increased firing rate of serotonergic neurons isolated from the ganglia, while within the ganglia, or even near it, their activity corresponds to a basic control level. Since application of dopamine antagonist sulpiride produced excitation only in rested after exercise neurons and only in the ganglia, it was suggested that dopamine volume signaling could be responsible for preventing overexcitation of PeA cells during the return to aquatic conditions.

Direct measurements of serotonin, its precursor and metabolites in the pedal and cerebral ganglia of *L. stagnalis* similarly revealed the profound differences in rested after exercise animals in comparison to the control, exercised for two or even for four hours, snails (Aonuma et al., 2020), pointing to the specific effect of rest after the exercise. Injections of serotonin or its precursor into snails reproduced the behavioral effects of intense locomotion only partially: both drugs decreased latency and increased locomotor activity in the arena, having no effect on the number of turns (Aonuma et al., 2020). These findings indicated the presence of seemingly conserved involvement of serotonin in the behavioral modulation by exercise. In vertebrates and invertebrates, serotonin is involved in modulation of cognitive functions and the central pattern generators for locomotion (Gillette, 2006; Kondo and Shimada, 2015).

## Insects

### Gryllus bimaculatus

Flight generates high energetic costs in insects (Harrison and Roberts, 2000). It is used for migrations, foraging, partner searching or rapid escape. In some insects, like crickets *G. bimaculatus*, flying occurs only in young animals preceding mating and serving to population dispersal (Dingle, 1972; Walker, 1986; Shiga et al., 1991; Lorenz, 2007). Interestingly, in the laboratory conditions, forced flying for 1–3 min produced profound changes in many behaviors of *G. bimaculatus*. First, it increased the aggressiveness in males. This influence was seen in socially naïve animals (Stevenson et al., 2005), as well as in male losers, in which flying restored the ability to fight the winner and even overcome him (Hofmann and Stevenson, 2000). Second, previous flying activated calling and courtship singing and resulted in faster mating success (Dyakonova and Krushinsky, 2008). It also decreased the escape response to the startling wind stimulation applied to the cricket cerci (Stevenson et al., 2005) In contrast, the response to male calling song was augmented during flying (Nolen and Hoy, 1986; Sergejeva and Popov, 1994) and 5 min after it (Mezheritskiy et al., 2020), Similarly, spontaneous activity and evoked responses to visual signals of motion-sensitive sensory neurons have been modulated by flying in the blowfly *Lucilia* spp., and octopamine agonists imitated the influence of flight (Jung et al., 2011). Therefore, while the avoidance response to disturbing tactile stimulation was decreased, the responses towards neutral or attractive sensory stimuli were potentiated by flight. Finally, female crickets after flying showed a better ability to find the way to reach the hidden source of the male call (Mezheritskiy et al., 2020). The latter finding suggests an augmentation of some cognitive skills by flying. The beauty of all these results is in their remarkable coherence. Indeed, all observed effects can be considered as promoting the competitiveness of animals (or their genes), in novel territories reached after flying.

The molecular mechanisms of flight induced behavioral modulation in crickets are poorly investigated. It is known that octopamine is secreted into the hemolymph during flight (Adamo et al., 1995). The agonists reproduce some of its behavioral effects, for instance the reset of aggressiveness in losers (Stevenson et al., 2005). On the other hand, octopamine produces effects that are opposite to those of flight, for example, it potentiates an avoidance in response to air puffs (Stevenson et al., 2000, 2005).

### *Drosophila* spp

Several different approaches have been developed to investigate the effects of exercise in *Drosophila melanogaster*, *D. simulans*, and other spp. (for recent review see Watanabe and Riddle, 2019). Unlike biologically-inspired neuroethological studies in *G. bimaculatus*, the majority of works in *Drosophila* spp. are translational medical-oriented research, in which the fly is considered as a model system for investigating the biochemical and genetic basis of exercise in humans. Nevertheless, the existing fly-exercising procedures are based on two naturally occurring forms of drosophila behavior, flight (Babcock and Ganetzky, 2014; Murashov et al., 2020) and vertical climbing, caused by negative geotaxis (Gargano et al., 2005; Sujkowski et al., 2015; Watanabe and Riddle, 2019). The effects of flying on feeding behavior and metabolism of wild-caught *Drosophila* spp. with learned preference for high fat, sugar, and salt were considered as beneficial (Murashov et al., 2020).

The effects of exercising in the vertical climbing paradigm have been significantly deeper and wider investigated. Regular climbing increased the life span (Wen et al., 2016), improved sleep in either old flies (Zheng et al., 2015) or flies with genetically induced Altzheimer phenotype, namely Aβ42-expressing flies (Berlandi et al., 2017). These effects however were absent in normal wild-type (Berlandi et al., 2017) or in flies selected for longevity (Sujkowski et al., 2015). Enhanced physical endurance, increased mitochondrial activity (Sujkowski et al., 2017; Damschroder et al., 2018), cardiac improvements (Piazza et al., 2009), increased lipolysis and reduced triglycerides, glycogen and weight (Wen et al., 2016) were also reported as benefits of exercise in *Drosophila* spp. which are similar to that in humans. Possibly conserved genes and metabolic pathways underlying these effects in mammals and flies have been searched for and found (Damschroder et al., 2018). For example, the similar role for TAFAZZIN Gene (taz) which encodes a protein that is expressed at high levels in cardiac and skeletal muscle was reported in flies and mice, so that Taz mutants in these species cannot improve endurance through exercise (Damschroder et al., 2018).

Both types of activities used in exercising flies (flight and vertical climbing) are known to activate the octopaminergic system (Ryglewski et al., 2017; Sujkowski et al., 2017). Indeed, octopamine and its receptors are necessary for improved endurance and some other effects of exercise in *Drosophila* spp. (Sujkowski et al., 2017, 2020; Cobb et al., 2020). At the same time, octopamine is a powerful neuromodulator affecting sensory (Farooqui, 2007) and cognitive (Giurfa, 2006) functions in insects. Surprisingly, we found no reports on exercise effects on cognitive skills in *Drosophila* spp. such as learning and memory, orientation or decision-making. This looks only as a temporal gap, as a number of neuronal genes and neuronal transcription regulators were reported recently to be affected by exercise in *Drosophila* spp. (Watanabe and Riddle, 2021).

## Discussion

Why and how the exercise-brain axis has been formed in human evolution has been discussed by anthropologists. Their ideas are based on specific human anatomy predisposed for long-term running (Bramble and Lieberman, 2004; Lieberman, 2012) and hunting activity of ancient humans which required combined physical and mental efforts (Raichlen and Alexander, 2017).

**Figure 1:**
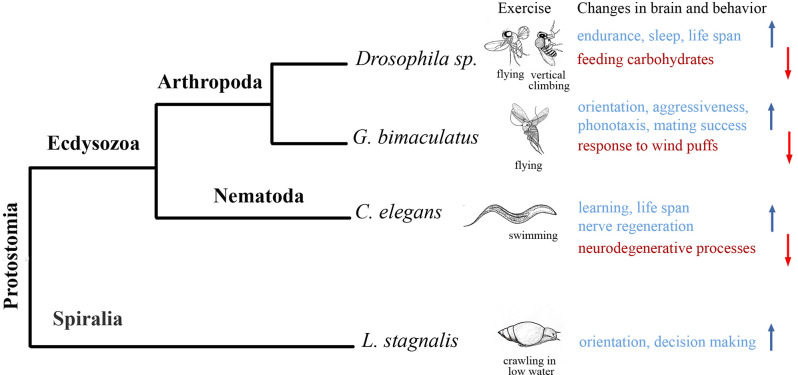
Effects of exercise (species-specific intense locomotion) on representatives of three big clades of Protostomes. Phylogenetic tree displaying the different species analyzed to study the effects of exercise on behavior and nervous system. The species are listed not in the order of appearance in the manuscript. Silhouettes are only illustrative. Species-specific forms of intense locomotion that were used as exercise are listed under the silhouettes. Exercise-induced changes in the brain and behavior are shown in color. Red color and down head arrow indicate downregulation, blue color and upward arrow indicate upregulation. In *Drosophila*, flying and or/vertical climbing increased endurance and life span, improved sleep and feeding behavior (for review, Watanabe and Riddle, 2019). In *G. bimaculatus*, flight improved orientation *via* conspecific phonotaxis (Sergejeva and Popov, 1994; Mezheritskiy et al., 2020), enhanced aggressiveness (Hofmann and Stevenson, 2000; Stevenson et al., 2005), promoted mating (Dyakonova and Krushinsky, 2008). In *C. elegans*, swimming protected against neurodegeneration, improved associative learning (Laranjeiro et al., 2019), accelerated nerve regeneration (Kumar et al., 2021). In *L. stagnalis*, previous terrestrial crawling increased activity and facilitated the decision making in novel environment (Korshunova et al., 2016).

In this review, we show that species-specific intense locomotion (exercise) improves brain functions in three big and distantly related clades of protostomes, namely Nematodes, Molluscs and Artropods (Figure 1). We do not know yet whether the aforementioned effects developed independently in these groups, as well as in protostomes and deutrostomes. The research of exercise-brain interactions in invertebrates has been started within the recent 7–8 years, and the data are still sparse. As such, definite conclusions to these speculations would be made prematurely. Many more organisms belonging to other taxa need to be investigated in this respect. Besides, different procedures were used and different effects of species-specific locomotion on cognitive functions were evaluated, which complicates the comparative analysis. In *C. elegans*, swimming protected against neurodegeneration, improved associative learning (Laranjeiro et al., 2019), accelerated nerve regeneration (Kumar et al., 2021); in *L. stagnalis*, terrestrial crawling increased activity and facilitated the decision making in novel environment (Korshunova et al., 2016); in *Drosophila* spp., vertical climbing increased endurance, improved sleep and feeding behavior (for review, Watanabe and Riddle, 2019), in *G. bimaculatus*, flight improved orientation *via* conspecific phonotaxis (Mezheritskiy et al., 2020), enhanced aggressiveness (Hofmann and Stevenson, 2000), promoted mating (Dyakonova and Krushinsky, 2008) and increased the resistance to disturbing stimuli (Stevenson et al., 2005). Many of these effects (underlined in the previous sentences) have been described in mammals as beneficial results of running, suggesting again certain similarity between distantly-related species. At the molecular and epigenetic level, clear similarities between mammals, insects and nematodes are seen in the regulation of metabolism by intense locomotion. In all three clades, exercise reduces glucose metabolism and increases fat metabolism affecting conserved metabolism-related genes. We believe that further exploration of mechanisms operating during exercise within nervous systems of various invertebrates will result in discovering a number of common pathways as well.

At the same time, this may impose a question: are studies in invertebrates something more than just a replication of works in rodents, have they implemented new knowledge? We believe that yes, they already have significantly enriched the field with novel findings, and seem to have even better perspectives.

The first lesson from invertebrate studies is that the exercise-brain phenomena is not unique to mammals, it is likely to have deep evolutionary roots. Could it have a biological sense common to different clades? Our original hypothesis posits that intense movement is interpreted by the organism as predictive of change, in anticipation of which adjustments are made. As a result, resources are mobilized towards increasing the speed and effectiveness of making decisions—the proverbial “think fast!”. Indeed, all behavioral effects of intense locomotion are favorable for adaptation and orientation in novel environments, as have been discussed in previous works on phylogenetically distant species (Korshunova et al., 2016; Aonuma et al., 2020; Mezheritskiy et al., 2020), including mammals. In the latter, exercise activates exploration of new territories (Yin et al., 2013), promotes new learning and forgetting old memories (Epp et al., 2016). Interestingly, in vertebrates and invertebrates exercise facilitates risk-taking and energetically expensive behaviors under uncertainty such as exploration (mammals), decision-making (mammals and snails), acoustic signaling, aggression and reproduction (insects). This may reflect a general homeostatic switch from stability to higher plasticity, seen at the behavioral, metabolic and genetic levels. The switch to a higher plasticity is an expected means of novelty preadaptation. At the same time, exercise effects in vertebrates and invertebrates are different from stress (Laranjeiro et al., 2017; Aonuma et al., 2020), and even abolish the consequences of several stressors (Heijnen et al., 2016).

Next interesting finding made in invertebrates suggests that, at least, in *C. elegans* and *Drosophila*, higher locomotor activity is linked to an increased life span in spite of its obvious energetic costs. Activating cognitive skills by physical exercise is additionally costly. Moreover, in the same species, higher cognitive skills and neuronal excitation are linked to decreased life span (Burger et al., 2008; Zullo et al., 2019; Dyakonova, 2020). However, intense locomotion (at least in some regimes), while having beneficial effects on cognitive performance, appears to have no negative effect on longevity. We suggest that this may be due to feedforward activation of antioxidant systems (already shown as exercise effect) and DNA reparation mechanisms (remain to be examined) to counterforce an increased plasticity of brain epigenome and to protect from higher risk of neuronal DNA mutations. The great perspective for future studies in invertebrate organisms is to search for the natural mechanisms mediating simultaneous plasticity and protection of CNS after exercise.

There are also some new insights into underlying cellular and molecular mechanisms which are made due to specific advantages of selected animal models. In *C. elegans*, the molecular mechanism for exercise-induced accelerated nerve regeneration at the single cell level is elucidated (Kumar et al., 2021). In *Lymnaea stagnalis*, changes in biophysical properties of isolated serotonergic neurons were discovered, as well as the involvement of volume neurotransmission in adaptation of the nervous system to physical exercise (Dyakonova et al., 2019). In *Drosophila* spp., in well controlled experiments with different regimes of training, the responsive neuronal genes and metabolic pathways are identified (Watanabe and Riddle, 2021); the role of octopaminergic system is evidenced in genetic, pharmacological and behavioral studies (Sujkowski et al., 2017, 2020; Cobb et al., 2020). In the cricket *G. bimaculatus*, the most complete repertoire of behavioral changes produced by flying have been described in neuroethological investigations on aggression (Hofmann and Stevenson, 2000), mating (Dyakonova and Krushinsky, 2008), auditory orientation (Sergejeva and Popov, 1994; Mezheritskiy et al., 2020). These studies allowed to describe flight-induced changes in terms of behavioral state and to formulate the first hypothesis on the biological significance of flight effect as possible adaptation to a novel environment (Stevenson et al., 2005; Dyakonova and Krushinsky, 2008; Mezheritskiy et al., 2020).

Investigations of the exercise-brain axis in the above organisms will most likely be continued. In each of them, certain gaps are evident either in methodological or theoretical terms. In molluscs, genetic approaches are missing, other cognitive skills and behaviors possibly affected by exercise are to be investigated. In *Drosophila* spp., there is no data on cognitive effects of exercise, the question on biological and ethological significance of climbing effects remains open. Crickets are insufficiently characterized in genetic and cellular experiments studying the underlying mechanisms. *C. elegans* has the intriguing perspectives for cellular and genetic analysis of swimming influence on learning and life span along with intrinsic protective mechanisms linking cognitive activity with prolonged life span. Besides, all invertebrate organisms would be useful for investigating possible intergenerational epigenetic effects of intense locomotion and their mechanisms. Until now, intergenerational effects of exercise have been reported only in rodents (for review, Yang et al., 2021), which are still less convenient models because of their relatively long life cycle.

## Author Contributions

VD and EI wrote the manuscript. MM analyzed literature on exercise effects in insects. TD and IC analyzed electrophysiological and cellular mechanisms in molluscs and *C. elegans*. IZ and DB prepared the figure and the drawings. All authors contributed to the article and approved the submitted version.

## Conflict of Interest

The authors declare that the research was conducted in the absence of any commercial or financial relationships that could be construed as a potential conflict of interest.

## Publisher’s Note

All claims expressed in this article are solely those of the authors and do not necessarily represent those of their affiliated organizations, or those of the publisher, the editors and the reviewers. Any product that may be evaluated in this article, or claim that may be made by its manufacturer, is not guaranteed or endorsed by the publisher.
